# Proteomic change in the upper lobe of the left lung of Beagle dogs at the lung migration stage of *Toxocara canis* infection

**DOI:** 10.1186/s13071-024-06302-9

**Published:** 2024-05-09

**Authors:** Wen-Bin Zheng, Hui-Jie Qiu, Han-Dan Xiao, Yang Zou, Xing-Quan Zhu

**Affiliations:** 1 Laboratory of Parasitic Diseases, College of Veterinary Medicine, Shanxi Agricultural University, Taigu, 030801 Shanxi People’s Republic of China; 2 State Key Laboratory for Animal Disease Control and Prevention, Key Laboratory of Veterinary Parasitology of Gansu Province, Lanzhou Veterinary Research Institute, Chinese Academy of Agricultural Sciences, Lanzhou, 730046 Gansu People’s Republic of China

**Keywords:** *Toxocara canis*, Dog, Toxocariasis, DIA, Proteomics

## Abstract

**Background:**

*Toxocara canis* is considered one of the most neglected parasitic zoonoses and threatens the health of millions of people worldwide with a predilection for pediatric and adolescent populations in impoverished communities. Exploring the invasion and developmental mechanisms associated with *T. canis* infection in its definitive canine hosts will help to better control zoonotic toxocariasis.

**Methods:**

Proteomic changes in samples from the upper lobe of the left lung of Beagle puppies were systematically analyzed by quantitative proteomic technology of data-independent acquisition (DIA) at 96 h post-infection (hpi) with *T. canis*. Proteins with *P*-values < 0.05 and fold change > 1.5 or < 0.67 were considered proteins with differential abundance (PDAs).

**Results:**

A total of 28 downregulated PDAs and 407 upregulated PDAs were identified at 96 hpi, including RhoC, TM4SFs and LPCAT1, which could be associated with the maintenance and repair of lung homeostasis. GO annotation and KEGG pathway enrichment analyses of all identified proteins and PDAs revealed that many lung proteins have correlation to signal transduction, lipid metabolism and immune system.

**Conclusions:**

The present study revealed lung proteomic alterations in Beagle dogs at the lung migration stage of *T. canis* infection and identified many PDAs of Beagle dog lung, which may play important roles in the pathogenesis of toxocariasis, warranting further experimental validation.

**Graphical Abstract:**

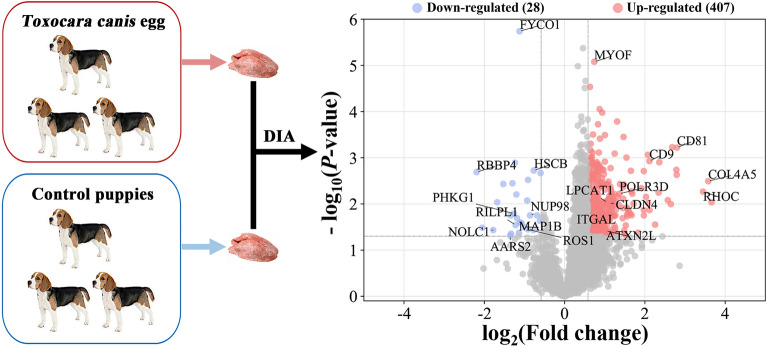

**Supplementary Information:**

The online version contains supplementary material available at 10.1186/s13071-024-06302-9.

## Background

Toxocariasis is one of the most prevalent parasitic zoonoses and is mainly caused by the roundworm *Toxocara canis*, afflicting millions of people worldwide with a predilection for pediatric and adolescent populations in impoverished communities, but it is neglected and underreported in many developing and developed countries [1, 2]. It is estimated that 11.1% of dogs have been infected with *T. canis* worldwide, and 19% of the world population was infected with* Toxocara* [3, 4]. A large number of *T. canis* eggs were shed by infected canines, such as coyotes, dogs, foxes and wolves, through the feces [1, 5], subsequently contaminating food, vegetables, water and public environment. For vegetables, cucurbits and environment soil samples, the pooled incidence of *Toxocara* egg contamination has been reported as 7%, 10% and 21%, respectively [6, 7], posing a severe risk to human and animal health.

*Toxocara canis* has a complex life cycle between paratenic and definitive hosts [8]. After embryonated eggs have hatched in host small intestine, L3 larvae penetrate the intestinal wall, enter the blood stream and reach the liver; subsequently, they are transported to the lung at 96 h post-infection (hpi), which is the lung infection period of *T. canis* in hosts [8]. In the lung, the L3 larvae begin to undergo two completely different migratory pathways in definitive and paratenic hosts [8]. In the definitive hosts, the L3 larvae can penetrate the pulmonary alveoli and reach the trachea, which is the prerequisite route for L3 larvae to re-enter the gastrointestinal tract, and the larvae can complete their development without causing severe damage to definitive canine hosts during the process of migration and development [8]. However, in humans or other paratenic hosts of animal species, the “lung migration stage” of *T. canis* infection is missing; although L3 larvae of *T. canis* can be found in lung tissues, they cannot arrive in the trachea through the lungs [9]. The larvae of *T. canis* cannot achieve their development in paratenic hosts, but they can migrate to several different tissues or organs in the host, causing severe pathological and debilitating clinical manifestations, such as visceral and ocular toxocariasis [10]. The lung appears to be the main organ affecting the differences in migration and developmental patterns of *T. canis* between the paratenic and definitive hosts [10]. This phenomenon is highly common among helminths of the genera *Toxocara* and *Ascaris* and raises an interesting question as to what role the lung plays in *T. canis* infection and its life cycle. The simple and reasonable speculation is that lung tissues, as the strategic anatomical location, play indispensable roles in responding to *T. canis* invasion.

Previous studies have shown that *T. canis* infection modulates the transcriptome and metabolome of Beagle dog lungs [11, 12], revealing the key roles of dog lungs in response to *T. canis* infection. However, the underlying molecular mechanism remains largely unclear. In recent years, LC–MS/MS-based high-throughput proteomics have become an essential technique for profiling proteomes and exploring molecular mechanisms of pathogenesis and disease resistance in body fluids, cells and tissues, as it provides information on protein signature, protein regulation and the signaling networks of specific diseases [13]. However, little is known about the proteomic change in the lung of Beagle dogs at the lung migration stage of *T. canis* infection.

Therefore, the present study analyzed proteomic alterations in the lung tissues of Beagle puppies infected with *T. canis* at 96 hpi by DIA-based quantitative proteomic approach to explore the potential molecular defensive mechanism of Beagle puppies in response to *T. canis* infection at the protein level. Proteomic characterization, proteins with differential abundance (PDAs), functions of the PDAs and key molecular pathways involved in response to *T. canis* infection between infected and healthy lung tissues of Beagle puppies were identified, screened, elucidated and predicted by multiple bioinformatics analyses.

## Methods

### Lung sample collection

Six 6- to 7-week-old Beagle dogs of both genders were purchased from the National Canine Laboratory Animal Resource Center (Guangzhou, China) and were divided into two groups: *T. canis*-infected group (*n* = 3) and control (*n* = 3) group. The dogs used in this study were handled in accordance with the laboratory animal-microbiological standards and monitoring (Standard id: GB 14922.2-2011). In addition, to ensure that each puppy was sero-negative for *T. canis* infection, anti-*T. canis* IgG antibodies were examined in the sera of the puppies by an indirect ELISA using larval excretory-secretory antigen, and feces from all puppies were examined by the sugar flotation method to ensure each puppy was free of gastrointestinal parasites according to our previous description [11]. Eggs of *T. canis* were collected from the uteri of fertile *T. canis* females which had been taken from dogs. The eggs were incubated on filter papers with 0.5% formalin solution at 28 °C with 85–95% relative humidity for 4 weeks. The infectious eggs were collected from the filter papers, filtered using 200-mesh screens and then stored in 1% formalin solution at 4 °C [11]. After the puppies had been fully acclimated to the environment for 1 week with water and food three times per day at 24 °C, each of the puppies in the *T. canis*-infected group was fed 300 embryonated *T. canis* eggs, and puppies in the control group were fed equal amounts of saline. At 96 hpi, puppies were humanely killed using Zoletil 50 (Virbac, Nice, Franch) with 10 mg/kg and 10% KCl with 0.75 mg/kg as previously described [11]. The lung tissues of the upper lobe of the left lung of the control puppies and *T. canis*-infected puppies were rapidly collected and frozen in liquid nitrogen, followed immediately by storing in a deep freezer at − 80 °C.

### Protein extraction and digestion

The protein extraction and digestion methods were performed according to previous description [14]; 20 mg lung tissue, 200 μl lysis buffer 3 (7 M urea, 2 M thiourea, 20 mM Tris–HCl, pH 8.0–8.5) with 0.2% SDS and 1 × Cocktail with EDTA were mixed in a 2-ml centrifuge tube and put on ice for 5 min. Then, the tissue was crushed by a grinder (60 HZ, 2 min) after adding two steel beads and DTT with the final concentration of 10 mM. Subsequently, supernatant was obtained after centrifuging at 25,000×*g* for 15 min at 4 °C, followed by being kept in a 56 °C water bath for 1 h. The mixed solution was placed in a dark room for 45 min after adding IAM at a final concentration of 55 mM. Then, cold acetone was added to the solution at a ratio of 1:5, and the mixture was placed in a refrigerator at − 20 °C for 30 min. Subsequently, the supernatant of the mixture was discarded after centrifuging as above. The protein pellet was air dried at 4 °C and was ground by a grinder (60 HZ, 2 min) after being dissolved in 300 μl lysis buffer 3 without SDS to promote pellet solubilization. Later, 250 μl supernatant was obtained by centrifuging as above, and the quality and concentration of the final protein solution were measured by Bradford quantification and SDS-PAGE. Then, 100 μg lung protein diluted with 50 mM NH4HCO3 by 4× volumes was digested with 2.5 μg trypsin at 37 °C for 6 h. Subsequently, all digested proteins were desalted by Strata X column (Phenomenex, Torrance, USA) and were vacuumed to dryness.

### Data-dependent acquisition (DDA) and DIA analysis

The methods of data-dependent acquisition (DDA) and DIA analysis were performed according to previous descriptions [14]. Digested proteins of 10 μg/sample were mixed and diluted by mobile phase A_1_ [5% acetonitrile solution (ACN), pH 9.8] and were subjected to the LC-20AB system (Shimadzu, Japan) with high-pH Gemini C18 column (5 µm, 4.6 mm × 250 mm; Waters, USA) to separate the liquid phase and to establish a reference library. This separation process was accomplished by regulating the mobile phase B_1_ (95% CAN, pH 9.8) with different concentration gradients at flow rate of 1 ml/min: 5% mobile phase B_1_ for 10 min, 5% to 35% mobile phase B_1_ for 40 min, 35% to 95% mobile phase B_1_ for 1 min, mobile phase B_1_ lasting 3 min and 5% mobile phase B_1_ equilibrated for 10 min. The elution peak was monitored at a wavelength of 214 nm, and component was collected every minute. Components were combined into a total of 10 fractions [14]. Then, each separated fraction was immediately freeze-dried. The peptide samples were re-dissolved with mobile phase A_2_ [2% ACN, 0.1% formic acid (FA)] and were enriched and desalted in the trap column (5 µm, 300 µm × 5 mm, Thermo Fisher Scientific, USA). Subsequently, they were subjected to U3000 ultra-high performance liquid chromatography (UHPLC) and were separated in the C18 column (1.8 μm, 150 μm × 35 cm, Thermo Fisher Scientific, USA) connected to a tandem mass spectrometer for subsequent DDA fractions and DIA samples analysis. This process was accomplished by regulating the mobile phase B_2_ (98% ACN, 0.1% FA) with different concentration gradients at a flow rate of 0.5 μl/min as previously described with appropriate modification [14]: 0–5 min, 5% mobile phase B_2_ (98% ACN, 0.1% FA); 5–130 min, mobile phase B_2_ linearly increased from 5 to 25%; 130–150 min, mobile phase B_2_ rose from 25 to 35%; 150–160 min, mobile phase B_2_ rose from 35 to 80%; 160–175 min, 80% mobile phase B_2_; 175–175.5 min, mobile phase B_2_ decreased from 80 to 5%; 175.5–180 min, 5% mobile phase B_2_ [14]. The nanoliter liquid phase separation end was directly connected to the mass spectrometer, and the separated peptides were ionized by nanoESI and subjected to a Fusion Lumos tandem mass spectrometer (Thermo Fisher Scientific, CA, USA) for DDA and DIA analysis.

In DDA detection mode, the main settings were: ion source voltage 2 kV; MS scan range 350–1500 m/z; MS resolution 60,000, maximal injection time (MIT) 50 ms; MS/MS collision type HCD, collision energy NCE 30; MS/MS resolution 15,000, MIT 50 ms and dynamic exclusion duration 30 s. The start m/z for MS/MS was fixed to 100. Precursors for MS/MS scan were: charge range 2+ to 6+, top 30 precursors with intensity over 2E4. Automatic gain control (AGC) was MS 3E6, MS/MS 1E5.

In the DIA detection mode, the main settings were: ion source voltage 2 kV; MS scan range 400–1500 m/z; MS resolution 60,000, MIT 50 ms; 400–1500 m/z was equally divided to 44 continuous window MS/MS scan. MS/MS collision type was HCD, MIT 54 ms. Fragment ions were scanned in Orbitrap, MS/MS resolution 30,000, collision energy 30; AGC was 5E4.

### Quantification and functional prediction of lung proteins

MaxQuant v.1.5.3.30 was used to identify the DDA data, and the main settings were as below: fixed modifications: carbamidomethyl (C); variable modifications: acetyl (protein N-term), deamidated (NQ), Gln- > pyro-Glu (N-term Q) and oxidation (M); enzyme: trypsin; minimal peptide length: 7; Database: *Canis lupus familiaris* downloaded from Ensembl database (44,816 sequences, CanFam3.1) and *T. canis* downloaded from WormBase ParaSite database (PRJNA248777, 18,596 sequences) [15]. The pollutant database, including human keratins (skin hair), bovine serum protein and yeast protein, was used in this study, and the common contaminants were filtered out during the analysis. The DIA data were analyzed using the iRT peptides for retention time calibration. Then, based on the target-decoy model applicable to SWATH-MS, false-positive control was performed with FDR 1%. In this study, MSstats software package was applied to intra-system error correction and normalization for each sample based on median, and then the proteins with differential abundance (PDAs) were identified based on the linear mixed effect model with the filtration criteria of *P*-values < 0.05 and fold change > 1.5 or < 0.67 [16]. The databases of Eukaryotic Orthologous Groups (KOG), Gene Ontology (GO) and Kyoto Encyclopedia of Genes and Genomes (KEGG) were used to predict the potential biological functions or pathways of identified proteins and PDAs.

## Results

### Quantification and functional prediction of lung proteins

**Fig. 1:**
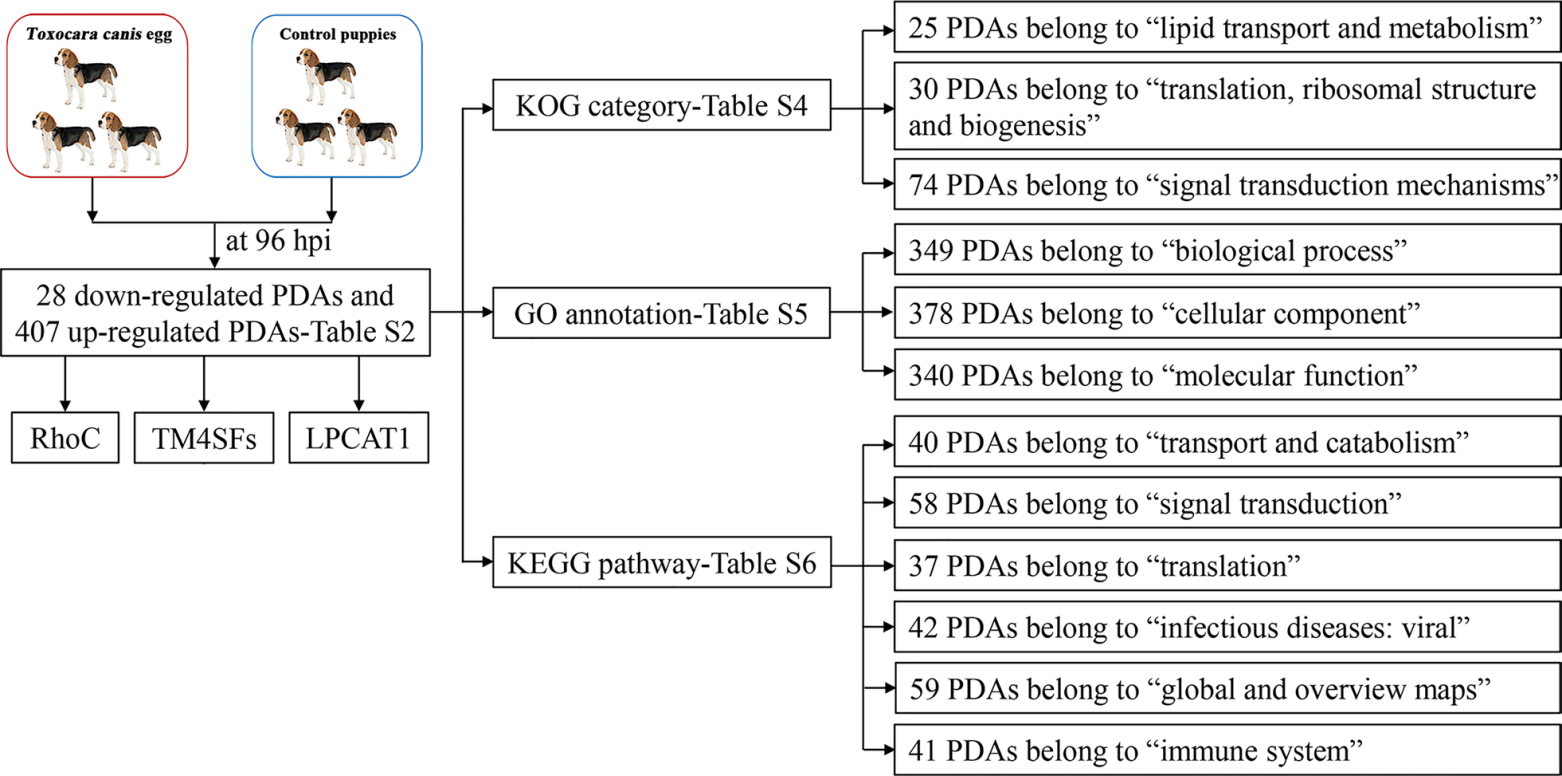
Workflow of data processing

**Fig. 2:**
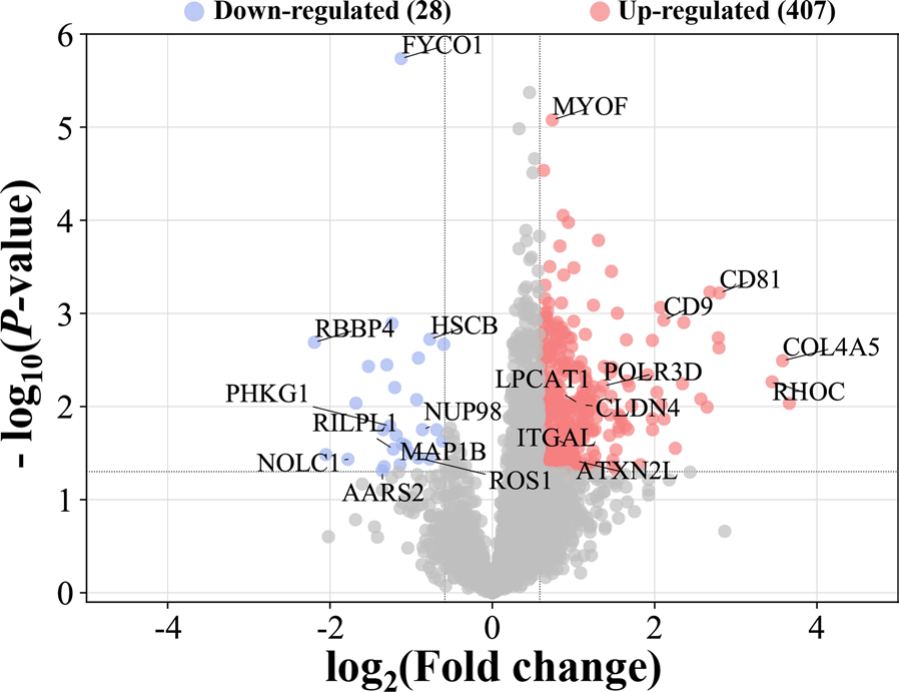
Volcano plots showing the proteins with differential abundance (PDAs) identified from puppies' lungs at 96 h after infection with 300 *Toxocara canis* eggs

**Fig. 3:**
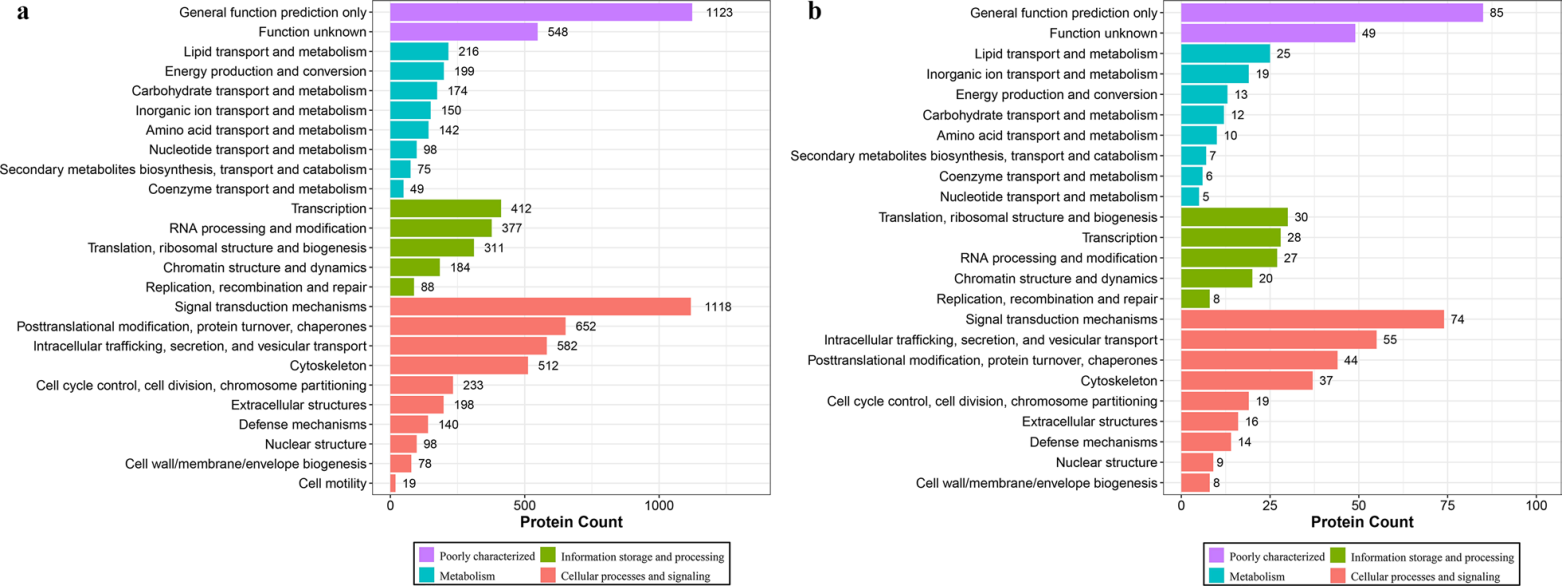
KOG enrichment analysis of all identified lung proteins (**a**) and lung proteins with differential abundance (**b**)

**Fig. 4:**
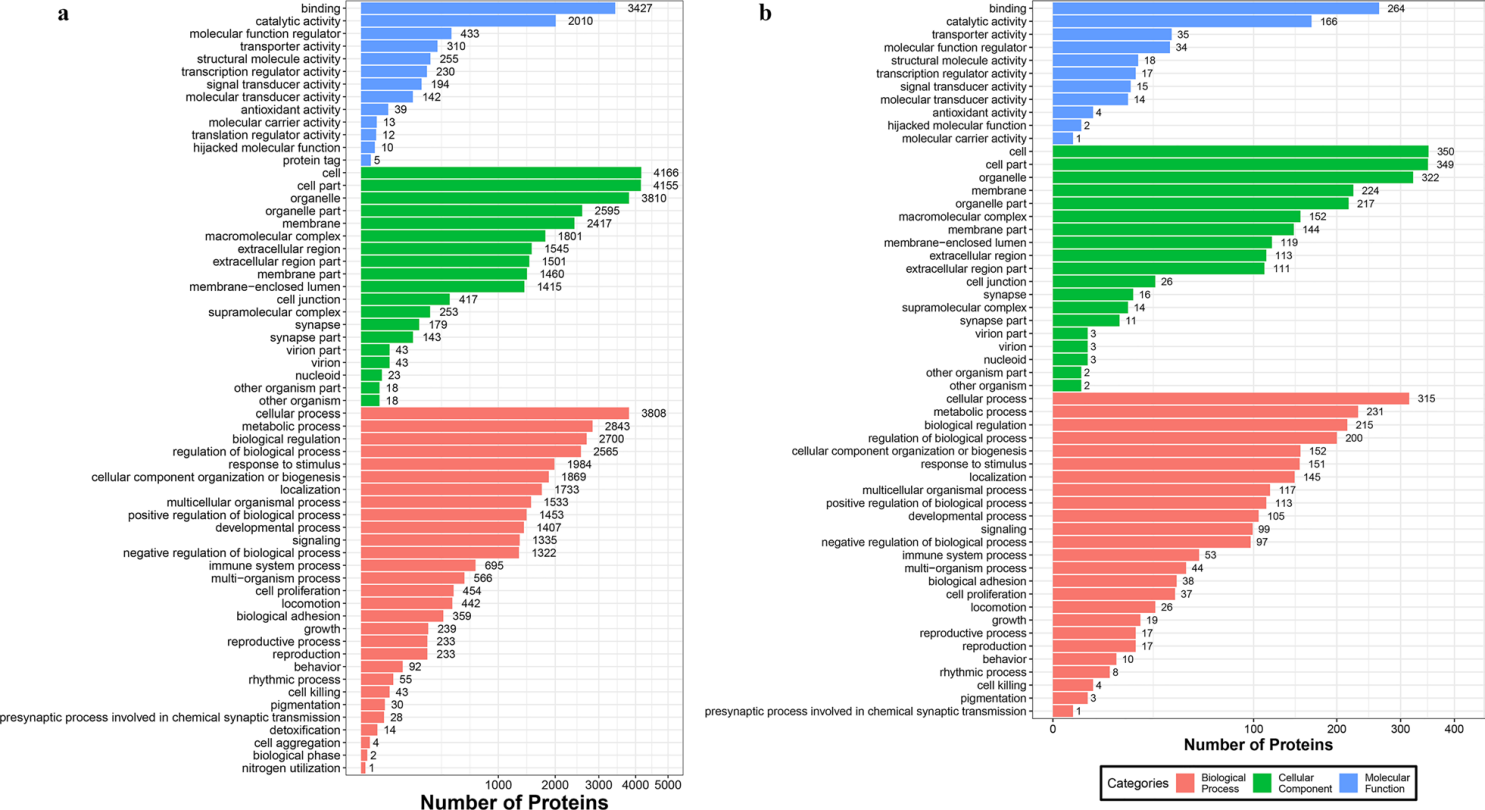
GO annotation of all identified lung proteins (**a**) and lung proteins with differential abundance (**b**), including biological process, cellular component and molecular function

**Fig. 5:**
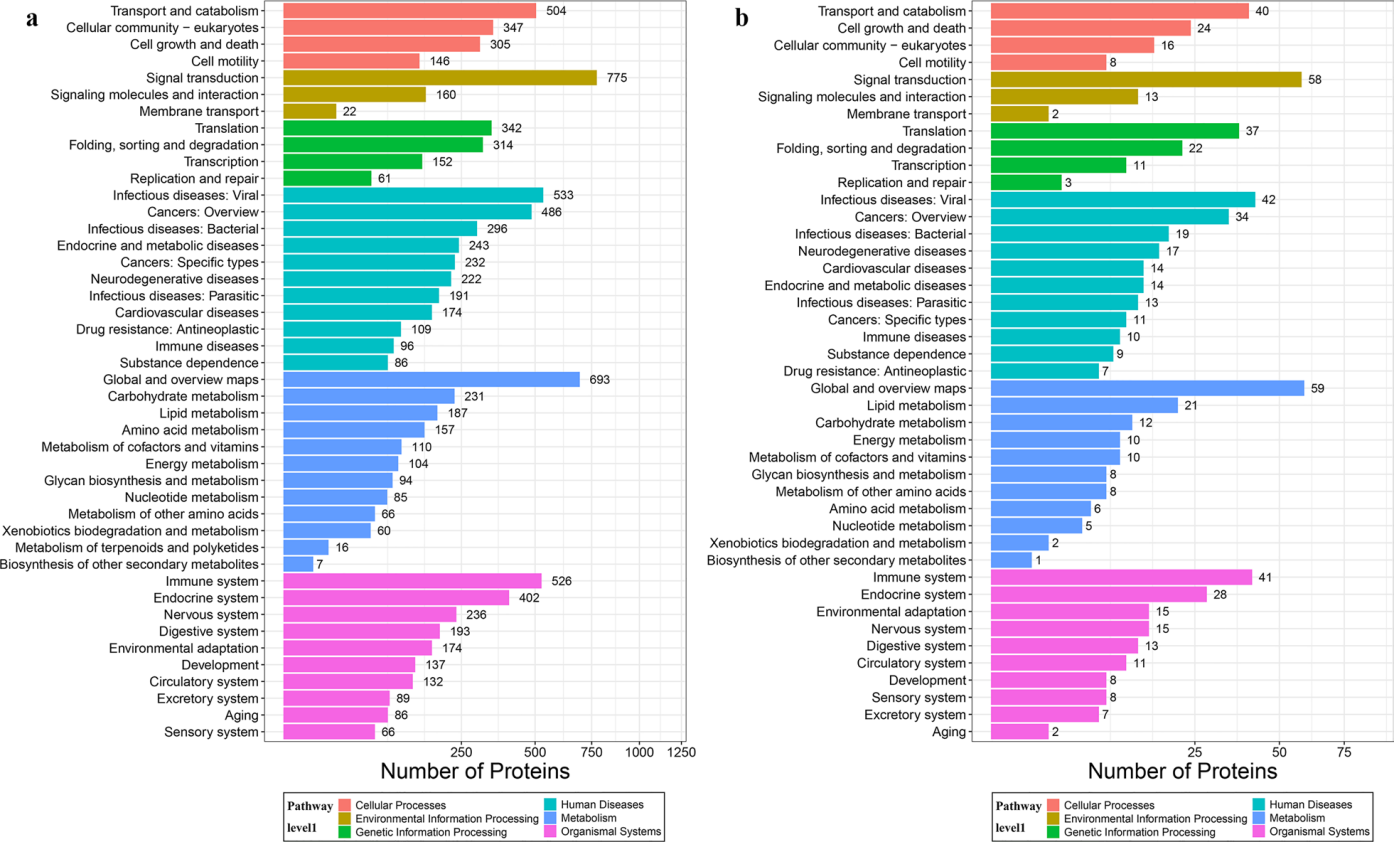
KEGG pathway enrichment analyses of all identified lung proteins (**a**) and lung proteins with differential abundance (**b**)

The workflow of data processing and associated outputs are shown in Fig. 1. A total of 41,150 peptides and 4957 lung proteins were identified at 96 hpi (Additional file 1: Table S1) with 28 downregulated PDAs and 407 upregulated PDAs, including RhoC, TM4SFs and LPCAT1 (Fig. 2 and Additional file 2: Table S2). To analyze whether contamination of somatic proteins or excretory-secretory products (ESPs) of *T. canis* existed in the selected portion of the lung, the proteome data were re-analyzed by combining both *T. canis* and *C. lupus familiaris* databases, and a total of 105 peptides and 53 potential *T. canis* proteins were identified with seven upregulated PDAs (Additional file 1: Table S1). This result also showed that most of these putative *T. canis* peptides and all seven putative upregulated PDAs of *T. canis* are present in both the infected and control groups, suggesting that there are no *T. canis* proteins. Among the 4957 identified lung proteins, 4825 were classified into 25 KOG categories. Among them, 548 proteins were of unknown function; “lipid transport and metabolism” was the largest functional category with 216 proteins in the family of “metabolism;” “transcription” was the largest functional category with 412 proteins in the family of “information storage and processing,” and “signal transduction mechanisms” was the largest functional category with 1118 proteins in the family of “cellular processes and signaling” (Fig. 3a and Additional file 3: Table S3); 4763 lung proteins were enriched in 61 GO items by GO annotation, including 4189 lung proteins in 29 GO items of “biological process,” 4507 lung proteins in 19 GO items of “cellular component” and 4154 lung proteins in 13 GO items of “molecular function” (Fig. 4a and Additional file 4: Table S4); 3258 lung proteins were enriched in 44 signaling pathways by KEGG pathway analysis; among these, “transport and catabolism” were highly enriched with 504 proteins in the item “cellular processes;” “signal transductions” were highly enriched with 775 proteins in the item “environmental information processing;” “translations” were highly enriched with 342 proteins in the item “genetic information processing;” “infectious diseases: viral” were highly enriched with 533 proteins in the item “human diseases;” “global and overview maps” were highly enriched with 693 proteins in the item “metabolism,” and “immune system” were highly enriched with 526 proteins in the item “organismal systems” (Fig. 5a and Additional file 5: Table S5).

### KOG, GO and KEGG analysis of PDAs

Among the 28 downregulated PDAs and 407 upregulated PDAs, 391 were classified into 24 KOG categories. Among them, 49 PDAs are of unknown function; “lipid transport and metabolism” was the largest functional category with 25 PDAs in the family of “metabolism;” “translation, ribosomal structure and biogenesis” was the largest functional category with 30 PDAs in the family of “information storage and processing,” and “signal transduction mechanisms” was the largest functional category with 74 PDAs in the family of “cellular processes and signaling” (Fig. 3b and Additional file 3: Table S3). Three hundred ninety-five PDAs were enriched in 55 GO items, including 349 PDAs in 25 GO items of “biological process,” 378 PDAs in 19 GO items of “cellular component,” and 340 PDAs in 11 GO items of “molecular function” (Fig. 4b and Additional file 4: Table S4). Two hundred sixty-five PDAs were enriched in 43 KEGG signaling pathways; among these, “transport and catabolism” were highly enriched with 40 PDAs in the item “cellular processes;” “signal transductions” were highly enriched with 58 PDAs in the item “environmental information processing;” “translations” were highly enriched with 37 PDAs in the item “genetic information processing;” “infectious diseases: viral” were highly enriched with 42 PDAs in the item “human diseases;” “global and overview maps” were highly enriched with 59 PDAs in the item “metabolism,” and “immune systems” were highly enriched with 41 PDAs in the item “organismal systems” (Fig. 5b and Additional file 5: Table S5).

## Discussion

Coyotes, dogs, foxes and wolves are common definitive hosts of *T. canis*, which contribute to the environmental egg burden. Among these, dogs usually have access to playgrounds and other public places, posing a tremendous risk of human exposure to infective eggs [1, 5]. In this study, aiming to uncover the protein response in the upper lobes of left lung samples of Beagle puppies infected with *T. canis*, we studied the global proteomic profile of Beagle dog lungs by DIA-based quantitative proteomics. A total of 4957 lung tissue proteins were identified among the 6 lung samples with 28 downregulated PDAs and 407 upregulated PDAs at 96 hpi in this study. Considering that the number of upregulated PDAs was much greater than that of downregulated PDAs, it is necessary to assess whether there is contamination of *T. canis* somatic proteins or ESPs in lung tissues. Thus, the proteome data were re-analyzed by combining both *T. canis* and *C. lupus* databases. Theoretically, if the lung tissue of *T. canis*-infected group used in this study contained *T. canis* somatic proteins or ESPs, many upregulated *T. canis* proteins would be identified in *T. canis*-infected puppies when the proteome data were re-analyzed by combining both *T. canis* and *C. lupus* databases instead of having just 7 upregulated PDAs (13.%, 7/53) and 46 not statistically different proteins. This result also showed that most of these putative *T. canis* peptides and all seven putative upregulated PDAs of *T. canis* were present in both infected and control puppies, suggesting that there are not protein components of *T. canis*. Therefore, this result proved that there are no somatic proteins or ESPs of *T. canis* in the lung tissue of infected puppies, and it is appropriate to use a single *C. lupus familiaris* database for the proteome data analysis.

In this study, the obvious change was that 58 PDAs were enriched in the “signal transduction” pathway, and the expression level of Ras homolog family member C (RhoC) was upregulated 10.91-fold (Fig. 5b and Additional file 5: Table S5). RhoC is an important component of intracellular signal transduction, and increasing evidence suggests that it has multiple biological functions, such as regulating actin organization, cytoskeletal reorganization and cellular motility [17, 18] and even participates in the development of multiple tumors and tumors stem cell therapeutics [19]. The overexpression of RhoC has been reported to be involved in regulating tumor cell proliferation, invasion and metastasis, including lung cancer [20, 21]. However, there has been no study on the biological function of RhoC in the response of hosts to worm infection. When mice were used as sepsis models, the mRNA and protein levels of Rho family were abnormally activated in lung, including ROCK1 and RhoC, whereas, as lung inflammation was controlled by treating with asperosaponin VI, the mRNA and protein levels of RhoC gradually returned to normal or further downregulation [22], which suggested that RhoC may be one of the indicators of lung inflammation. The abnormal upregulation of 10.91-fold RhoC protein level may suggest that inflammation could occur in the lung tissues of puppies induced by *T. canis* infection.

The L3 larvae of *T. canis* which were passively transported with blood in the pulmonary vein at 96 hpi can penetrate the alveolar wall, invade the trachea and then reach the throat [8]. In this process, it is bound to cause a certain degree of damage to lung cells, but no obvious clinical symptoms were observed in all infected puppies at 96 hpi [11]. Another obvious change is that the expression level of many members of the transmembrane 4 superfamily (TM4SF) was upregulated in this study, such as CD9, CD63, CD81 and CD151. All these proteins were classified into “poorly characterized” category by KOG analysis in this study (Additional file 3: Table S3), suggesting that the biological function of these TM4SF proteins could not been fully elucidated; whereas all these proteins were enriched in many GO items, such as “membrane part,” “regulation of biological process,” “response to stimulus” and “localization” by GO annotation analysis. Moreover, CD9 was enriched in “immune system;” CD63 was enriched in “transport and catabolism” and “cancers: overview;” CD81 was enriched in “infectious diseases: parasitic,” “infectious diseases: viral” and “immune system” by KEGG pathway analysis (Additional file 4: Table S4 and Additional file 5: Table S5). These results suggested that these PDAs of TM4SF were involved in various cellular processes and have multiple biological functions. Their localization on the cell membrane indicates that they have a wide variety of biological activities in the interaction between cells and the extracellular matrix, such as cell adhesion, differentiation growth, motility, metastasis and signal transduction [23, 24]. TM4SFs play important roles in maintaining host function and are vital to control diverse inflammation and modulate humoral immune responses [25, 26]. For example, CD9 is a negative regulator of LPS-induced lung inflammation [27]. CD9 and CD81 are two widely distributed and closely correlated TM4SFs, which can play preventive roles in systemic inflammation of chronic obstructive pulmonary disease (COPD) [28]. In this study, the expression levels of CD9 and CD81 were upregulated 4.32-fold and 6.96-fold, respectively. Previous studies have revealed that the upregulation of CD81 could alleviate the LPS-induced injury in A549 cells, and the reduction of CD9/CD81 may be associated with the progression of inflammatory lung diseases, especially in COPD [29, 30]. CD9/CD81 knockout mice are more susceptible to developing emphysema and atrophy of various organs, including muscle, thymus and testis, and even shorten survival [31]. The upregulation of CD9 induced by statin treatment can decrease lung inflammation [32]. A previous study of lung transcriptomics indicated that the pathogenesis of toxocariasis was mediated through contributions from pro- and anti-inflammatory mechanisms [11]. Although there was no study on the role of these proteins in the response of hosts to helminth infection, we speculated that some TM4SFs could play important roles in reducing the lung inflammatory response induced by *T. canis* infection and maintaining host homeostasis. In addition to the TM4SF proteins, the expression levels of other transmembrane proteins, such as CLDN4, CD34 and CD46, were upregulated 2.15-, 6.89- and 2.95-fold, respectively, in this study. The biological functions of these transmembrane glycoproteins in the interaction between host and *T. canis* deserve further research.

A previous study showed that 88 lipid species were significantly altered in the puppies’ lungs at 96 h after *T. canis* infection [12]. The pathway of “lipid metabolism” was highly enriched with 21 PDAs in the item “metabolism” in this study, such as LPCAT1, EHHADH and PLA2G4A. Lysophosphatidylcholine acyltransferase 1 (LPCAT1) is an enzyme that catalyzes the biosynthesis of surfactant lipids and is expressed substantially in the lungs, especially in type 2 alveolar epithelial cells, and it catalyzes the synthesis of dipalmitoyl-phosphatidylcholine (PC), which is the major component of pulmonary surfactant [33–35]. The roles of dipalmitoyl-PC are to decrease the surface tension for proper alveolar opening and to protect lung cells from oxidative stresses [36]. LPCAT1 deficiency can promote pulmonary emphysema through apoptosis of alveolar epithelial cells, suggesting that LPCAT1 plays an important role in cell survival [37]. The expression level of LPCAT1 was upregulated 2.09-fold in this study. Although there was no study on the biological function of LPCAT1 in the response of hosts to helminth infection, it is reportedly upregulated in several carcinomas and is associated with cancer cell proliferation and the alteration of lipid composition [38]. A previous study showed that 12 different types of PC lipid species were altered with 3 upregulated and 9 downregulated in the puppies’ lungs at 96 h after *T. canis* infection [12]. Therefore, we speculated that the upregulation of LPCAT1 and alteration of PC lipid species could contribute to the renewal and repair of alveolar cells after *T. canis* infection.

A previous RNA-seq analysis showed that the biological functions of dog PBMCs may be comprehensively suppressed at 96 h after *T. canis* infection; however, 21 mRNAs in ECM-receptor interaction pathway were upregulated significantly at 96 hpi, including integrin genes, ECM constituents and proteoglycan [39]. In this study, we found that the integrin protein of ITGAL and ITGA4 were upregulated 1.92- and 1.84-fold, respectively, and the collagen of COL4A5, COL18A1 and COL11A1, key components of the ECM, were upregulated 11.93-, 1.91- and 1.86-folds, respectively. The invasion of *T. canis* larvae in the lungs of puppies can cause lung damage by both physical and immunological effects. Therefore, the upregulation of lung mRNAs and proteins in ECM-receptor interaction pathway may be attributed to the repair of the lung damage. Besides, many proteins involved in mRNA synthesis, gene expression and protein modification were significantly upregulated, such as POLR3D, CEBPB, ATXN2L and PLCB1, whereas some proteins associated with histone-binding and nucleoporins were significantly downregulated, such as RBBP4, NOLC1 and NUP98. The function of these PDAs in the pathogenesis of toxocariasis needs to be explored and elucidated.

The lung is an important organ during the migration and development of *T. canis*; however, only three biological replications in the upper lobe of the left lung of puppies were tested in this study, which was the limitation to reveal the migration of *T. canis* and explore the potential molecular defensive mechanism of Beagle puppies in response to *T. canis* infection within the whole lung. The alteration of spatial transcriptome and proteome of the lung infected with *T. canis* should be explored in future research to reveal the role of the lung in the migration and development of *T. canis* by including more animals.

## Conclusions

The present study examined the proteomic changes in the upper lobe of left lung at the lung migration stage of *T. canis* infection by DIA-based quantitative proteomic approach in samples from Beagle dogs. The results revealed that the number of upregulated PDAs was greater than that of the downregulated PDAs at 96 hpi, and many upregulated PDAs were associated with the maintenance and repair of lung homeostasis. GO annotation and KEGG pathway enrichment analyses of all identified proteins and PDAs revealed that many lung proteins have a correlation to signal transduction, lipid metabolism and immune system. This study provides new insights into the molecular defensive mechanism of Beagle dogs' response to *T. canis* infection by proteomic approach, suggesting that many lung proteins of puppies play important roles in the pathogenesis of *T. canis* infection in definitive canine hosts.

### Supplementary Information

**Additional file 1: Table S1.** Overview of all the identified peptides and proteins in lung samples of Beagle puppies.

**Additional file 2: Table S2.** Proteins with differential abundance and in the lungs of Beagle dogs infected by *Toxocara canis*.

**Additional file 3: Table S3.** KOG enrichment analysis for all identified proteins and the proteins with differential abundance.

**Additional file 4: Table S4.** GO functional annotation analysis for all identified proteins and the proteins with differential abundance.

**Additional file 5: Table S5.** KEGG enrichment analysis for all identified proteins and the proteins with differential abundance at the pathway level 2.

## Data Availability

The mass spectrometry proteomics data have been deposited at the ProteomeXchange Consortium (http://proteomecentral.proteomexchange.org) via the iProX partner repository [40, 41] with the dataset identifier PXD041668.

## Abbreviations

hpi: Hours post-infection
LC-MS/MS: Liquid chromatography-tandem mass spectrometry
PDAs: Proteins with differential abundance
ESPs: Excretory-secretory products
RhoC: Ras homolog family member C
TM4SF: The transmembrane 4 superfamily
COPD: Chronic obstructive pulmonary disease
LPCAT1: Lysophosphatidylcholine acyltransferase 1
PBMCs: Peripheral blood mononuclear cells
